# Pathological β-Cell Endoplasmic Reticulum Stress in Type 2 Diabetes: Current Evidence

**DOI:** 10.3389/fendo.2021.650158

**Published:** 2021-04-22

**Authors:** Neha Shrestha, Elisa De Franco, Peter Arvan, Miriam Cnop

**Affiliations:** ^1^ Department of Molecular and Integrative Physiology, University of Michigan Medical School, Ann Arbor, MI, United States; ^2^ Institute of Biomedical and Clinical Science, University of Exeter College of Medicine and Health, Exeter, United Kingdom; ^3^ Division of Metabolism, Endocrinology & Diabetes, Department of Internal Medicine, University of Michigan Medical School, Ann Arbor, MI, United States; ^4^ ULB Center for Diabetes Research, Medical Faculty, Université Libre de Bruxelles, Brussels, Belgium; ^5^ Division of Endocrinology, Erasmus Hospital, Université Libre de Bruxelles, Brussels, Belgium

**Keywords:** endoplasmic reticulum, stress, unfolded protein response, insulin, PERK (PKR-like endoplasmic reticulum kinase), ATF6 (activating transcription factor 6), IRE1 (inositol-requiring enzyme 1)

## Abstract

The notion that in diabetes pancreatic β-cells express endoplasmic reticulum (ER) stress markers indicative of increased unfolded protein response (UPR) signaling is no longer in doubt. However, what remains controversial is whether this increase in ER stress response actually contributes importantly to the β-cell failure of type 2 diabetes (akin to ‘terminal UPR’), or whether it represents a coping mechanism that represents the best attempt of β-cells to adapt to changes in metabolic demands as presented by disease progression. Here an intercontinental group of experts review evidence for the role of ER stress in monogenic and type 2 diabetes in an attempt to reconcile these disparate views. Current evidence implies that pancreatic β-cells require a regulated UPR for their development, function and survival, as well as to maintain cellular homeostasis in response to protein misfolding stress. Prolonged ER stress signaling, however, can be detrimental to β-cells, highlighting the importance of “optimal” UPR for ER homeostasis, β-cell function and survival.

## Overview

Pancreatic islet β-cells are specialized secretory cells designed for massive insulin storage. β-cells have a huge biosynthetic capability, which is further upregulated in insulin resistant states. The endoplasmic reticulum (ER) is the major organelle responsible for secretory protein synthesis, folding and quality control. To maintain ER homeostasis during the stresses associated with secretory protein synthesis and folding, cells activate the intracellular transduction pathway termed the unfolded protein response (UPR) (1).

**Figure 1:**
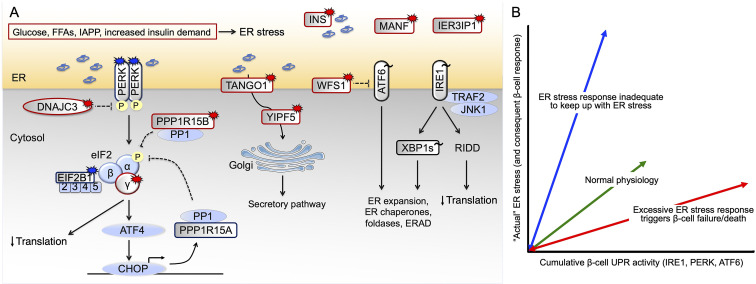
Pathways leading to β-cell ER stress and failure. **(A)** Genetic and non-genetic causes of human β-cell ER stress in diabetes. Patients with *EIF2AK3* and *EIF2B1* mutations that result in impaired signaling downstream of PERK develop young-onset diabetes, as do patients with *EIF2S3, DNAJC3* and *PPP1R15B* mutations that result in excessive PERK signaling. Homozygous *WFS1*, *YIPF5*, *TANGO1, MANF* and *IER3IP1* mutations also cause excessive UPR. Burst symbol indicates loss-of-function mutation causing monogenic diabetes, with red color indicating excessive and blue insufficient UPR signaling. For GWAS variants associated with T2D risk (indicated by tilde), the direction of effect is most often not known (black color). **(B)** Potential dysregulated β-cell responses to nutritional/metabolic challenge. UPR function is required for normal β-cell physiology, sustaining β-cell health and survival (green arrow). In conditions of insufficient UPR signaling relative to the prevailing ER stress (blue arrow), β-cells will fail. Conversely, excessive UPR signaling will cause β-cell dysfunction and death (red arrow). Currently available data provide evidence for both red and blue scenarios taking place in β-cells in T2D. ERAD ER-associated degradation, FFAs free fatty acids, IAPP islet amyloid polypeptide, RIDD regulated IRE1-dependent decay.

The UPR signals through three branches involving PERK, ATF6 and IRE1 (**Figure 1**) (2). PERK phosphorylates eIF2α, thereby attenuating protein translation and activating transcriptional responses mediated by transcription factors ATF4, CHOP and ATF3, with downstream induction of PPP1R15A and -B to dephosphorylate phosho-eIF2α, thereby limiting overshoot. ATF6 upregulates chaperones (including BiP) and foldases. IRE1 splices XBP1 mRNA to synthesize an active transcription factor that induces ER folding enzymes and chaperones, ER size expansion, and ER-associated degradation (ERAD). IRE1’s endoribonuclease activity also degrades ER-localized mRNAs in a process called regulated IRE1-dependent decay (RIDD), thereby indirectly decreasing translational demand placed upon the organelle. Besides their role in ER stress response, the tripartite limbs of the UPR play important roles in proinsulin production, folding and trafficking as well as β-cell expansion (3, 4).

Despite the critical roles of UPR in promoting β-cell function, sustained and unresolved UPR signaling (i.e., exuberant ER stress response) can be detrimental to β-cells. Chronic PERK activation increases expression of proapoptotic C/EBP homologous protein (CHOP, also known as growth arrest and DNA-damage inducible-153, GADD153) (5). Chronic IRE1 activation can lead to the recruitment of TRAF2 with subsequent activation of proapoptotic JNK and MAPK pathways. Prolonged IRE1 activation may also promote apoptosis through RIDD (6).

Perspectives of the UPR are thus divided between whether UPR signaling is actually beneficial or harmful for β-cell function. Accumulating studies suggest that both too much and too little UPR is detrimental to β-cells, highlighting the importance of UPR-mediated ER homeostasis. Here, we cite examples of monogenic diabetes, type 2 diabetes (T2D) genome-wide association studies (GWAS) and non-genetic triggers of diabetes to provide evidence both for and against UPR influences in β-cell physiology and T2D pathogenesis. We also discuss some caveats of ER stress hypotheses in establishing cause-and-effect on β-cell dysfunction.

## Monogenic Forms of Diabetes Highlight β-Cell Sensitivity to UPR Dysregulation, Suggesting That a Fine-Tuned ER Stress Response Is Needed for Maintenance of Functional β-Cell Mass

Monogenic diabetes is caused by highly penetrant single gene mutations, with the unique potential to reveal genes necessary for human β-cell development, function and survival. Of >40 genes with mutations reported to cause diabetes, 11 directly or indirectly result in β-cell ER stress. Discussing all of them would be beyond the scope of this review; here we consider several that reveal specific mechanisms by which dysregulation of ER stress affects human β-cells (**Figure 1**).

Mutations in the *WFS1* gene, encoding Wolframin (7), are known to result in a spectrum of phenotypes including β-cell dysfunction and diabetes. Wolframin has multiple proposed roles in β-cells, including in insulin production, processing and secretion, regulation of ER calcium levels, and suppression of ER stress-mediated cell death. Biallelic *WFS1* loss-of-function mutations result in childhood-onset diabetes [median age of onset 6 years, range 1-32 (8)], with dysregulated β-cell UPR, plus other phenotypes including optic atrophy, deafness, and diabetes insipidus. Heterozygous *WFS1* mutations may also be linked to isolated presentation of each of these phenotypes as well as isolated nuclear cataracts, with distinct genotype-phenotype relationships (9, 10). Recently, *de novo* heterozygous *WFS1* mutations were identified as causing a neonatal syndrome characterized by diabetes with onset in the first months of life and other congenital features (9). Functional *in vitro* studies suggest that these *de novo WFS1* mutations cause protein aggregation accompanied by a strong, dysregulated ER stress response and likely cell death (9), highlighting a separate disease mechanism from WFS1 loss-of-function.

Other ER-resident proteins implicated in monogenic diabetes include mesencephalic astrocyte-derived neurotrophic factor (*MANF*) (11) and immediate early response 3 interacting protein 1 (*IER3IP1*) (12); they may directly or indirectly modulate the UPR.

Genetic defects in at least 5 genes regulating the UPR through the PERK branch cause early onset β-cell failure and diabetes. Biallelic mutations in the *EIF2AK3* gene (encoding PERK) and dominant mutations in *EIF2B1* (resulting in loss of sensitivity of the EIF2B complex to eIF2α phosphorylation) result in syndromic forms of neonatal/early onset diabetes and likely β-cell death (13, 14). In these two diseases, signaling downstream of PERK is impaired. Recessive loss-of-function mutations in *EIF2S3* (15), *DNAJC3* [encoding a BiP co-chaperone (16)] and *PPP1R15B* [encoding a constitutive repressor of eIF2α phosphorylation (17)] all result in syndromic early-onset monogenic diabetes subtypes with excessive signaling downstream of PERK. These findings highlight β-cell sensitivity to perturbations of eIF2α phosphorylation and regulated mRNA translation in response to ER stress (18).

Recent studies suggest that ER stress caused by impaired ER-to-Golgi trafficking is another pathway resulting in monogenic diabetes. Recessive *YIPF5* missense mutations have been reported in patients with neonatal diabetes, microcephaly and epilepsy (19). Functional studies showed that β-cells are specifically sensitive to YIPF5 loss-of-function, increasing ER stress and apoptosis. Complete YIPF5 loss-of-function (by CRISPR/Cas9 knockout) resulted in proinsulin retention and β-cell ER stress, but did not affect α-cells (19). In keeping with this, a homozygous loss-of-function mutation in *TANGO1*, encoding an ER transmembrane protein that plays an essential role in ER-to-Golgi transport of bulky cargo, was reported in one family with syndromic diabetes (20).

Collectively, these monogenic forms of diabetes highlight the sensitivity of human β-cells to dysregulation of ER stress response or ER-to-Golgi trafficking, and are consistent with the notion that β-cell dysfunction may result from either an inadequate or an excessive ER stress response (21).

## GWAS of T2D Identify Variants in Genes Essential for ER Stress Regulation

In more complex and common diseases such as T2D, clues to the genetic and physiological mechanisms may be inferred from large-scale GWAS. To date, >400 genetic loci have been associated with T2D risk (22). Some of these are in proximity of UPR genes such as the well-established *WFS1* risk variants (23) that are associated with reduced insulin secretion (24). Of these, the *WFS1* rs10010131 intronic variant is traditionally recognized as the variant characterizing the linkage block in this region, which includes both coding and non-coding variants (23). However, over 10 years after their initial genetic discovery, it is still unclear which variant primarily causes the T2D risk, highlighting the challenges that remain to define the biological mechanisms underlying genetic predisposition.

Variants in *ERN1* (encoding IRE1α) exhibit a strong genetic association with T2D, possibly caused by dysfunctional alleles or diminished IRE1 protein expression. *XBP1* and *ATF6* exhibit a weaker association with T2D (25). *ATF6* variants have been suggested to be associated with increased T2D risk in East-Asians (26, 27). An upstream variant in *XBP1* has been suggested to be associated with increased T2D risk, increased fasting plasma glucose levels and fasting insulin levels in Han Chinese (28). Two common variants in the *CREBRF* gene, encoding a transcriptional repressor of the UPR, have been recently found to be associated with obesity and T2D risk in Pacific Highlanders (29). Variants in *PDX1* have a strong genetic association with random and fasting glucose (25). Interestingly, besides its role in β-cell development (30), PDX1 also maintains ER health through regulation of the ER calcium pump SERCA (31, 32).

These data highlight defects in ER stress regulation as a potential biological mechanism contributing to T2D predisposition, but additional studies are needed to confirm this hypothesis. Of course, T2D is a complex disease that develops as a result of the interplay of “lifestyle” (e.g., diet, exercise) with a myriad of genetic variants — thus it is unrealistic to expect that a single β-cell stress response can explain the spectrum of human T2D.

## Non-Genetic Triggers of β-Cell ER Stress in T2D Provide Evidence for the Importance of Subthreshold ER Stress in β-Cell Function

*In vitro* and *in vivo* studies have shown that β-cells activate the UPR following exposure to high glucose (33, 34); fatty acids - especially when saturated (35, 36); increased functional demand (as in the context of insulin resistance) (37, 38); or insulin and islet amyloid polypeptide (IAPP) misfolding/aggregation (39–41) (**Figure 1**).

With regards to nutrient stimulation, high glucose induces a moderate ER load, thereby activating subthreshold ER stress that drives insulin biosynthesis through IRE1 (42) as well as β-cell proliferation through ATF6 (34). Saturated free fatty acids, on the other hand, induce sustained activation of IRE1, PERK and ATF6 pathways (35, 43), resulting in β-cell apoptosis. Misfolded proinsulin in the ER lumen triggers the UPR to enhance protein folding and degradation but in case of *INS* mutations (causing mutant INS-gene-induced diabetes of youth, MIDY), the misfolding continues, resulting in persistent activation of the UPR (44). In such cases, β-cell apoptosis may be seen, most likely triggered by the transcriptional activation of CHOP (45). Finally, increased insulin demand also increases expression of UPR and chaperone proteins as exemplified by animal models of obesity (*ob/ob* mice) and T2D (*db/db*) (38). The increase in UPR in these models may ultimately be both beneficial and maladaptive. The UPR in these models of increased insulin demand is frequently upregulated during β-cell expansion/adaptation phases (34, 38, 46–48) to promote β-cell proliferation, yet the further benefit to β-cell mass and insulin secretion seen upon CHOP deletion in *db/db* models seems to diminish what would otherwise be a detrimental UPR contribution (49).

Crucially, ER stress may be triggered in β-cells during the development and/or after the onset of T2D in a vicious circle in which ER stress will impair β-cell function while the metabolic consequences may amplify the stress. The relative importance of ER stress in the chicken or egg dilemma may vary between different T2D patients considering the heterogeneity of disease pathogenesis. It is conceivable that in some patients, ER stress caused by genetic or environmental risk begets β-cell failure, while in others it is the hyperglycemia, dyslipidemia and increased insulin demand that begets β-cell ER stress.

Studies of human T2D include ultrastructural evidence of ER volume expansion in β-cells compared to non-diabetic control β-cells, suggesting response to ER stress (50). Although BiP and sXBP1 mRNAs were found to be decreased in islets of T2D individuals, they were induced in T2D islets upon high glucose exposure (50). However, in the largest RNA-sequencing study of human islets from T2D vs non-diabetic organ donors published to date (n=28 vs 58), no significant differences in expression of these genes have been detected (36). Engin et al. reported decreased sXBP1, ATF6, and P-eIF2α immunostaining in T2D islets compared with non-diabetic controls (46). Increased immunostaining for BiP, DNAJC3, ATF3 and CHOP was observed in human islets from T2D patients compared with islets from non-diabetic individuals; some of the staining was β-cell-specific (39, 51, 52). Clearly, more studies are needed on human islets from T2D patients, such as the transplantation of human islets from non-diabetic and T2D organ donors in immunocompromised mice subsequently exposed to high fat diet (53, 54), in order to longitudinally follow ER stress, function and survival of human β-cells in real time. Importantly, while these studies show correlative evidence to suggest changes in the total levels of β-cell UPR-related proteins, no studies have been able to report the bioactivity of these proteins (and thus the biological significance of these changes). Thus, much work is needed to determine whether, *in vivo*, exuberant ER stress response contributes to β-cell death in T2D.

## Caveats of the “ER Stress Hypothesis”

ER stress response is often cited as a double-edged sword which, upon chronic or excessive signaling, triggers apoptosis (55, 56). While *in vitro* studies clearly support these ideas, there is generally a lack of direct evidence to support this hypothesis *in vivo*. It may be that *in vivo*, signaling crosstalk within and among different organelles, and within and among different cells, allows cells and tissues to cope with and survive chronic ER stress in ways that are not easily clarified from *in vitro* studies. Indeed, we note that a sustained high level of UPR signaling is often not observed *in vivo*, unlike most *in vitro* studies that tend to use large acute doses of non-physiological ER stressors. Importantly, UPR pathways upregulate expression of ERAD and autophagy genes for degradation of misfolded proteins — in turn, ERAD (in particular the Sel1L-Hrd1 complex) can downregulate protein expression of IRE1α *via* its degradation, thereby limiting excessive UPR signaling (57). Another study showed that β-cells have endogenous mechanisms involving N-myc interactor and UbiquitinD to provide negative feedback signal on IRE-induced JNK activation in response to cytokines (58, 59). WFS1 is yet another stress-induced gene with a potential to inhibit ATF6 and tone down the UPR (60). These studies point to the very real possibility that excessive UPR signaling may be more restricted *in vivo*. Indeed, recent single cell analysis showed that healthy β-cells constantly undergo cycles of high and low UPR signaling, highlighting the importance of UPR dynamics in normal β-cell physiology (61), which is certainly not contrary to the notion that increased UPR activity during diabetes development represents a physiological response that may provide overall benefit to β-cells.

The human β-cell capital is probably fixed by young adulthood and human β-cells age with the body (62, 63). Unlike in type 1 diabetes, β-cell death is generally not considered a major player in the initial onset of T2D, which is primarily characterized by β-cell dysfunction (64) and even dedifferentiation (65). In fact, many studies have now demonstrated that ER dysfunction in β-cells may lead to dedifferentiation irrespective of ER stress response. For instance, dysfunction of ERAD induced by β-cell-specific genetic deletion of Sel1L, results in rapid and severe β-cell dedifferentiation. Importantly, these effects appeared independent of UPR, and instead altered the expression of TGFβ receptors — the activity of which promotes β-cell de-differentiation (66). Indeed, several studies have now shown that loss of insulin expression followed by dedifferentiation in cultured human β-cells is associated with induction of epithelial-mesenchymal transition-related pathways (67, 68). These studies point to the idea that signals emanating from the ER may directly contribute to β-cell failure by alternative mechanisms to that of apoptosis. In fact, IRE1 (which, as noted above, is an essential component of the UPR) also appears to play an important role in β-cell differentiation, as shown in a recent study (69) in which deletion of *Ire1* in NOD mice prior to insulitis resulted in β-cell dedifferentiation that could potentially help to limit antigen presentation to the immune system.

## Conclusions

The levels of UPR signaling in β-cells normally shift back and forth, which is likely to be important for proinsulin biosynthesis and folding during the metabolic/nutritional fluctuations that represent normal physiology. However, many studies show correlation of increased UPR and ER stress-associated genes with β-cell dysfunction and apoptosis. The data are particularly convincing from *in vitro* studies with pharmacologic ER stressors, as well as with high glucose and/or fatty acid exposure. However, animal models and a large number of human monogenic forms of diabetes (and to some extent, human GWAS) support that it is a dysregulated ER stress response that is more likely to predispose to diabetes onset, suggesting that a functional, homeostatic ER stress response is generally β-cell protective *in vivo*. Thus our consensus view is that UPR function is required for normal β-cell health and survival, although it may go awry in conditions of excessive or unresolvable ER stress or dysregulated signaling (**Figure 1**). Other aspects of ER homeostasis, beyond the UPR, may be equally (or even more) important in determining β-cell phenotype(s) in T2D. Ultimately, a systematic and detailed study of the time-course of UPR signaling during different stages of β-cell dysfunction in T2D will be needed in order to provide greater clarity about the role(s) of ER stress response/UPR in the β-cell failure of “garden-variety” T2D, and the subclassifications that will continue to emerge from a growing understanding of personalized medicine.

## Author Contributions

All authors: conceptualization. MC: investigation, PA, NS, EF, MC: writing—original draft, and PA, NS, MC: writing—review and editing. PA, NS, EF, MC: resources and funding acquisition. PA and MC: supervision. All authors contributed to the article and approved the submitted version.

## Funding

PA is supported by NIH R01 DK48280, and R01 DK111174. EF is a Diabetes UK RD Lawrence Fellow (19/0005971). The MC lab is funded by the Fonds National de la Recherche Scientifique (FNRS), the Fonds Erasme for Medical Research, the Brussels Region Innoviris project DiaType, the Walloon Region SPW-EER Win2Wal project BetaSource, the Francophone Foundation for Diabetes Research (FFRD, that is sponsored by the French Diabetes Federation, Abbott, Eli Lilly, Merck Sharp & Dohme and Novo Nordisk) and the Innovative Medicines Initiative 2 Joint Undertaking Rhapsody, under grant agreement No 115881, supported by the European Union’s Horizon 2020 research and innovation programme, EFPIA and the Swiss State Secretariat for Education, Research and Innovation (SERI) under contract number 16.0097.

## Conflict of Interest

The authors declare that the research was conducted in the absence of any commercial or financial relationships that could be construed as a potential conflict of interest.

## Abbreviations

ER, endoplasmic reticulum; ERAD, ER-associated degradation; GWAS, genome-wide association studies; MIDY, mutant INS-gene-induced diabetes of youth; RIDD, regulated IRE1-dependent decay; T2D, type 2 diabetes; UPR, unfolded protein response.
